# Human-AI Collaboration in Proximal Early Caries Lesion Detection: Potential Benefits for Novices and Potential Risks for Experts

**DOI:** 10.1159/cre/abuag009

**Published:** 2026-09-10

**Authors:** Ricardo E. Gonzalez-Valenzuela, Quoc T.D. Vu, Pascal Mettes, Bruno G. Loos, Henk Marquering, Erwin Berkhout

**Affiliations:** a Department of Oral Radiology, Academic Centre for Dentistry Amsterdam (ACTA), University of Amsterdam and Vrije Universiteit Amsterdam, Amsterdam, The Netherlands; b ACTA AI Lab, Academic Centre for Dentistry Amsterdam (ACTA), University of Amsterdam and Vrije Universiteit Amsterdam, Amsterdam, The Netherlands; c Department of Biomedical Engineering and Physics, Amsterdam University Medical Center (AUMC), University of Amsterdam and Vrije Universiteit Amsterdam, Amsterdam, The Netherlands; d VISlab, Informatics Institute, University of Amsterdam (UvA), Amsterdam, The Netherlands; e Department of Periodontology, Academic Centre for Dentistry Amsterdam (ACTA), University of Amsterdam and Vrije Universiteit Amsterdam, Amsterdam, The Netherlands; f Department of Radiology and Nuclear Medicine, Amsterdam University Medical Center (AUMC), University of Amsterdam and Vrije Universiteit Amsterdam, Amsterdam, The Netherlands

**Keywords:** Artificial intelligence, Bitewing radiography, Dental caries detection, Self-reported confidence

## Abstract

**Introduction:**

Early-stage proximal caries detection on bitewing radiographs is challenging and hampered by inter-observer variability. This study aimed to assess how artificial intelligence (AI) support influences radiographic detection and staging of proximal early caries lesions, confidence, and assessment time across observer experience levels.

**Methods:**

Eighteen observers (dental students, general dentists, and dental radiologists) evaluated 369 proximal surfaces on 25 bitewing radiographs in two sessions: first unaided, then aided with AI-annotated caries lesion regions. Observers assigned lesion stages and provided self-reported (SR) confidence ratings. Radiographic detection and staging accuracy relative to an expert consensus reference standard was assessed using cumulative link mixed models. Ordinal agreement and binary detection metrics were computed for each observer group.

**Results:**

The AI-based tool had divergent effects across the different groups of observers. Dental students showed a statistically significant improvement in radiographic detection and staging accuracy relative to the reference standard (*p* = 0.039). General dentists and dental radiologists showed no statistically significant change in radiographic detection and staging accuracy when using AI support (*p* = 0.28 and *p* = 0.15, respectively). SR confidence increased when the AI-based tool was used for all groups.

**Conclusion:**

AI support induced a shift in observers’ detections toward the AI-based tool’s performance pattern, with its effect on radiographic detection and staging accuracy varying across observer groups and no statistically significant change observed in more experienced observers. AI generally increased the SR confidence of the clinicians.

## Introduction

Proximal caries lesion assessment relies heavily on the interpretation of bitewing radiographs, yet early proximal lesions often appear as faint or ambiguous radiolucencies that are frequently overlooked [1]. The subtle appearance of early caries lesions leads to notable interpretive inconsistency among clinicians, with inter-observer variability repeatedly documented in radiographic caries-detection research [2].

In recent years, artificial intelligence (AI), especially convolutional neural networks, has been applied to dental image analysis with increasing frequency [3]. According to a comprehensive review, AI systems demonstrate high performance in caries detection, and the authors emphasized their potential value as instruments that can strengthen accuracy and aid clinical decision-making [4, 5]. Therefore, several investigations have explicitly proposed AI-based tools as supportive aids for oral health professionals, particularly to enhance interpretive consistency, reduce perceptual errors, and assist in treatment-related decision-making [6].

In controlled trials, the impact of AI on clinician behavior has been examined. For instance, a randomized trial in general dentists assessing bitewing radiographs showed that AI support led to a clear improvement in sensitivity [7]. Meanwhile, another randomized study using eye-tracking showed that dentists with AI annotations exhibited more efficient viewing behavior, more fixations on teeth with restorations or caries, shorter time to first fixation, and less time invested on healthy teeth, indicating that AI may alter how clinicians visually search and extract information from radiographs [8–10].

Although recent studies have applied AI to proximal caries assessment, several important limitations remain. First, evidence from International Caries Classification and Management System (ICCMS)-oriented models shows that classifying lesions into multiple categories is technically challenging and that current AI models struggle particularly with early-stage enamel involvement [11, 12]. Second, other recent studies indicate that AI models may support initial detection and help standardize image interpretation, yet questions about generalizability persist because most available datasets remain relatively homogeneous [13, 14]. Third, these studies primarily quantify algorithmic performance and do not examine how AI support influences observers’ radiographic detection and staging accuracy relative to a reference standard and their self-reported (SR) confidence. Together, these limitations confine current evidence for AI to proximal caries assessment largely to algorithmic detection performance. As a result, little is known about how AI-support influences clinicians’ radiographic assessment across lesion stages and datasets where clinical assessment is most challenging.

To enable a systematic investigation of how an AI-based tool influences clinical decision-making in proximal early-caries assessment, we developed the ACTA-Bw25-RefStd dataset. This publicly available collection comprises bitewing radiographs paired with expert-consensus proximal early caries lesion stage and treatment labels, providing a robust reference standard for early proximal caries assessment. Using this resource, we examined AI-supported decision-making across oral health professionals with different levels of expertise (dental students, general dentists, and dental radiologists), focusing on how AI support may affect radiographic detection and staging accuracy relative to the reference standard and their SR confidence. The aim of this study was to evaluate whether AI support affects oral health professionals’ radiographic detection and staging accuracy for proximal early caries lesion diagnosis relative to the reference standard, as well as their SR confidence.

## Materials and Methods

### Data

#### Dataset Selection

A total of 25 bitewing radiographs were selected, by dental radiologists, from a large, anonymized educational and research repository maintained at the Department of Oral Radiology at the Academic Centre for Dentistry Amsterdam (ACTA). These 25 bitewing radiographs constitute the ACTA-Bw25-RefStd dataset and are publicly available. Inclusion criteria included bitewings containing permanent dentition and proximal surfaces that were either radiographically sound or predominantly presented enamel or outer-dentin radiolucencies; a small number of deeper dentin radiolucencies were also present. Exclusion criteria included bitewing radiographs of insufficient diagnostic quality, defined as images with inadequate contrast, excessive noise, motion artifacts, or positioning errors that precluded reliable assessment of proximal surfaces.

#### Image Acquisition

All selected bitewing radiographs from the ACTA-Bw25-RefStd dataset were obtained using a wall-mounted Focus intraoral X-ray unit (KaVo Dental, Biberach, Germany) equipped with a long-cone rectangular collimator. The system operated at 60 kV and 7.0 mA, with an exposure time of 0.20 s. Images were acquired using a size-2 Gendex GXS700 digital intraoral sensor (KaVo Dental) and exported as 16-bit TIFF files.

#### Image Viewing and Analysis

All annotation and evaluation sessions, including reference standard creation and study-participant assessments, were performed under controlled viewing conditions. Sessions took place in a darkened room using a calibrated Dell U2419H monitor (24-inch, 1,920 × 1,080 px, 60 Hz, SDR). Calibration followed an SMPTE test pattern, and brightness and contrast were standardized at 75% to ensure consistent luminance and contrast perception across all observers and sessions. Caries lesion stages were recorded using a radiographic scoring scale aligned with ICCMS radiographic categories: 0 = sound (RA0), 1 = enamel radiolucency (RA1–RA2), 2 = outer dentin radiolucency (RA3), and 3 = deeper dentin radiolucency (RB4–RC6).

#### Reference Standard Creation

A total of 369 proximal surfaces were examined across the 25 bitewing radiographs. Three dental radiologists, each with over 10 years of clinical experience, jointly reviewed all images on a shared workstation and scored by consensus. They are designated the “expert panel” and produced the reference standard by assigning, for each proximal surface, its corresponding caries lesion stage (0–3) [15].

The ACTA-Bw25-RefStd dataset also includes caries lesions area delineations (represented as binary mask images where 0 = background and 1 = lesion), generated by the same expert panel. A separate bounding-box table lists the *x*_1_, *y*_1_, *x*_2_, *y*_2_ coordinates for all 369 proximal surfaces. Although these bounding boxes were not used in the present study, they were included to enhance clarity, transparency, and usability for readers and further studies.

#### Observer Groups Description

A total of 18 dental professionals participated, divided into three observer groups: six final-year dental students, six general dentists, and six dental radiologists. The groups were balanced in gender (three females and three males per group). Professional experience differed across groups: with dental students in their final year of training, general dentists had a median experience of 6.5 years (IQR: 2–11; range: 1–15), and dental radiologists with a mean experience of 8.0 years (IQR: 2–11; range: 4–14). All observers independently assessed every proximal surface in both sessions. Observer characteristics are provided in online supplementary Material A (for all online suppl. material, see https://doi.org/10.1159/cre/abuag009).

#### Participants Annotation Protocol

To investigate the impact of AI assistance on radiographic detection and staging judgments, participants carried out two consecutive evaluation sessions: an initial unaided assessment followed by a second session incorporating the output of an AI-based tool. The tool used was dentalXrai Pro (version 3.0.12.3; dentalXrai Ltd., Berlin, Germany), which displays visual markers highlighting algorithm-predicted proximal caries lesions.

Across both sessions, participants reviewed the same set of 25 bitewing radiographs and documented, for each of the 369 proximal surfaces, the caries lesion stage (0–3) [15] and their SR-confidence level (0 = not at all sure, 1 = only a little sure, 2 = fairly sure, 3 = quite sure, and 4 = definitely sure). Observers were explicitly instructed to detect and stage only putative caries-related lesions; defects not related to a suspected caries process were not to be classified as lesions. Every observer evaluated all proximal surfaces.

Treatment recommendations were collected to explore whether AI-supported radiographic detection influenced downstream decision-making. The methods and results of this analysis are reported separately in online supplementary material B.

To minimize potential sources of bias, three methodological measures were implemented. First, a washout period of at least 4 weeks separated the two sessions to reduce recall of prior evaluations. Second, AI support was provided only in the second session to ensure that participants’ initial judgments remained uninfluenced by algorithmic suggestions. Finally, the bitewing radiographs were presented in a different order in the second session to further limit memory effects, while maintaining the same order for all participants within the two sessions.

No time limit was imposed on participants in either session; however, each was to be completed in a single continuous sitting. Any brief interruptions, if they occurred, were excluded from the recorded time. Timing started when the participant began assessing the first bitewing radiograph and ended upon completion of the final assessment. Radiographs were evaluated using ImageJ software, which allowed observers to freely adjust image brightness and contrast during the assessment, as commonly done in clinical image interpretation.

#### Data Analytic Tools

All analyses and computations were carried out using a combination of R (version 4.5.2) and Python (version 3.14.0). In R, we relied on the ordinal, lme4, irr, tidyverse, and boot packages for ordinal modeling, mixed-effects inference, agreement statistics, data manipulation, and bootstrap-based uncertainty estimation. Python was used for data preprocessing and dataset management, employing libraries such as pandas, numpy, scikit-learn, matplotlib, and seaborn.

#### Statistical Analysis

Radiographic caries lesion staging was analyzed using cumulative link mixed models, which are well suited for handling ordinal data and for accommodating nested data structures through random effects [16]. Each proximal surface served as the observational unit. To reflect the structure of the data, the models included random intercepts for observers, acknowledging that each observer evaluated the same proximal surfaces twice, once without and once with AI support. In addition, random intercepts were included for proximal surfaces nested within bitewings, capturing the dependence among surfaces that originate from the same radiograph. Additional information on power and sample size estimation is provided in online supplementary Material C.

To answer our research question – Does AI support improve radiographic caries lesion detection and staging accuracy relative to the reference standard? – the analysis did not directly compare the raw lesion stages assigned in session 1 and session 2. Rather, the goal was to compare how well each session’s accuracy relative to the reference standard. For every proximal surface, we quantified the number of staging category steps by which the observer-assigned stage differed from the stage indicated by the reference standard. Lesion stages were treated as ordered categories, and the distance was defined as the absolute difference between the observer-assigned stage and the reference stage (e.g., a difference of 0 for an exact match, 1 if the stages differed by one category, and so on). This distance was then transformed into an agreement score, with higher values representing more accurate stage assignment relative to the reference standard. A perfect match received the highest score, and the score decreased as the observer’s staging diverged further from the reference standard.

Using this agreement score as the outcome, we used the CLMM method to evaluate whether the distribution of detection accuracy scores differed between sessions with and without AI support. For each observer group, as well as for all observers combined, a full model including the session term was compared with a null model without the AI-supported session term using a likelihood-ratio test. From these models, odds ratios and 95% confidence intervals (CIs) were derived, indicating whether radiographic detection and staging accuracy relative to the reference standard improved or declined when AI support was available.

#### Performance Metrics

Performance metrics for caries lesion detection and staging were evaluated at the group level (dental students, general dentists, and dental radiologists) under three approaches. First, we compared the observer performance in the session without AI support with the reference standard. In a second analysis, observer performance in the session with AI support was compared with the reference standard. The third performance analysis was the intra-observer approach, in which performance from the session without AI support was compared with performance from the session with AI support, for each observer group. Additionally, the same metrics were computed for the standalone AI system, allowing direct comparison with observer groups, and between humans and the AI tool across the three approaches.

Quadratic-weighted Cohen’s κ was used to quantify ordinal agreement with the reference standard for caries lesion stage assignments. κ values were computed for each observer group under all three approaches. To characterize uncertainty in group performance, bootstrap resampling of observers was performed independently within each professional group using 1,000 iterations. In every bootstrap iteration, observers were resampled with replacement from the original group (*n* = 6 per group), κ values were recalculated, and percentile-based 95% CIs were obtained, capturing between-observer variability while preserving the crossed reading structure. Extended observer-level agreement methods and results are available in online supplementary Material D.

Moreover, binary detection metrics were computed for dichotomized lesion-stage outcomes. These included sensitivity, specificity, balanced accuracy, positive predictive value (PPV), negative predictive value (NPV), and F1 score. Metrics were obtained for each observer group under all three approaches, and the same set of metrics was computed for the standalone AI system to provide a consistent point of reference for comparing human performance with the AI output. Because the AI tool produced lesion regions rather than ordinal staging, a member of the “expert panel” assigned a lesion stage to each AI-detected region. Supplementary binary detection metrics, including alternative thresholds and observer-level results, are provided in online supplementary Material E.

To enable an integrated overview of the performance metrics, radar (spider) plots were generated for the without-AI and with-AI sessions. Each plot simultaneously displays the full set of performance metrics allowing direct visual comparison of multidimensional performance profiles across observer groups and the AI-based tool. This approach highlights relative strengths and weaknesses within each group and makes shifts induced by AI support immediately interpretable.

#### SR-Confidence Patterns across Lesion Stages

To compare SR confidence between correct and incorrect assessments across lesion stages, we generated composite bar-and-line plots for each observer group and for all observers combined. For each session (with and without AI support), mean SR confidence and corresponding 95% CI were calculated separately for correct and incorrect stage assignments. Bar graphs display mean values with CI, while superimposed lines connect the stage-specific means to highlight overall trends.

#### Session-Duration Analysis

To examine whether AI support reduced the time required to complete the assessment, session durations with and without AI support were recorded and compared using paired Wilcoxon signed-rank tests. Analyses were conducted per observer group and for the full sample.

## Results

### Reference Standard Creation

Table 1 presents the results for the reference standard allocated treatment decisions across lesion stage categories. Sound surfaces were entirely classified as requiring no action. Enamel caries lesions were predominantly assigned to monitoring, with fewer cases placed under no action or restoration. Outer dentin caries lesions were mainly associated with restorative treatment, followed by monitoring. Inner dentin caries lesions were exclusively assigned to restoration. No cases in any lesion stage category were allocated to fluoride-only treatment.

**Table 1.** Reference standard outcomes linking lesion-stage categories to treatment decisions for a total of 369 proximal sides

Stage\treatment (total, *n* = 369 sites)	No action	Monitoring	Fluoride-only	Restoration
Sound (*n* = 217), *n* (%)	217 (100)	–	–	–
Enamel caries (*n* = 96), *n* (%)	7 (7)	87 (91)	–	2 (2)
Outer dentin caries (*n* = 52), *n* (%)	–	17 (33)	–	35 (67)
Inner dentin caries (*n* = 4), *n* (%)	–	–	–	4 (100)

Values represent the number of occurrences followed by the corresponding percentage per lesion-stage category.

Figure 1 presents an example of a bitewing radiograph from the ACTA-Bw25-RefStd dataset with the consensus reference-standard annotations. Carious regions identified by the reference standard are delineated with red contours. Each proximal surface is marked by a bounding box, with blue representing distal surfaces and green representing mesial surfaces. The text placed within each bounding box reports two reference-standard labels: (1) the radiographic depth category (D), indicating the extent of the carious involvement assigned to that surface, and (2) the recommended treatment (T) associated with that surface. These elements together summarize the reference-standard outcome used for lesion-level and surface-level analyses in subsequent evaluations.

**Fig. 1.**
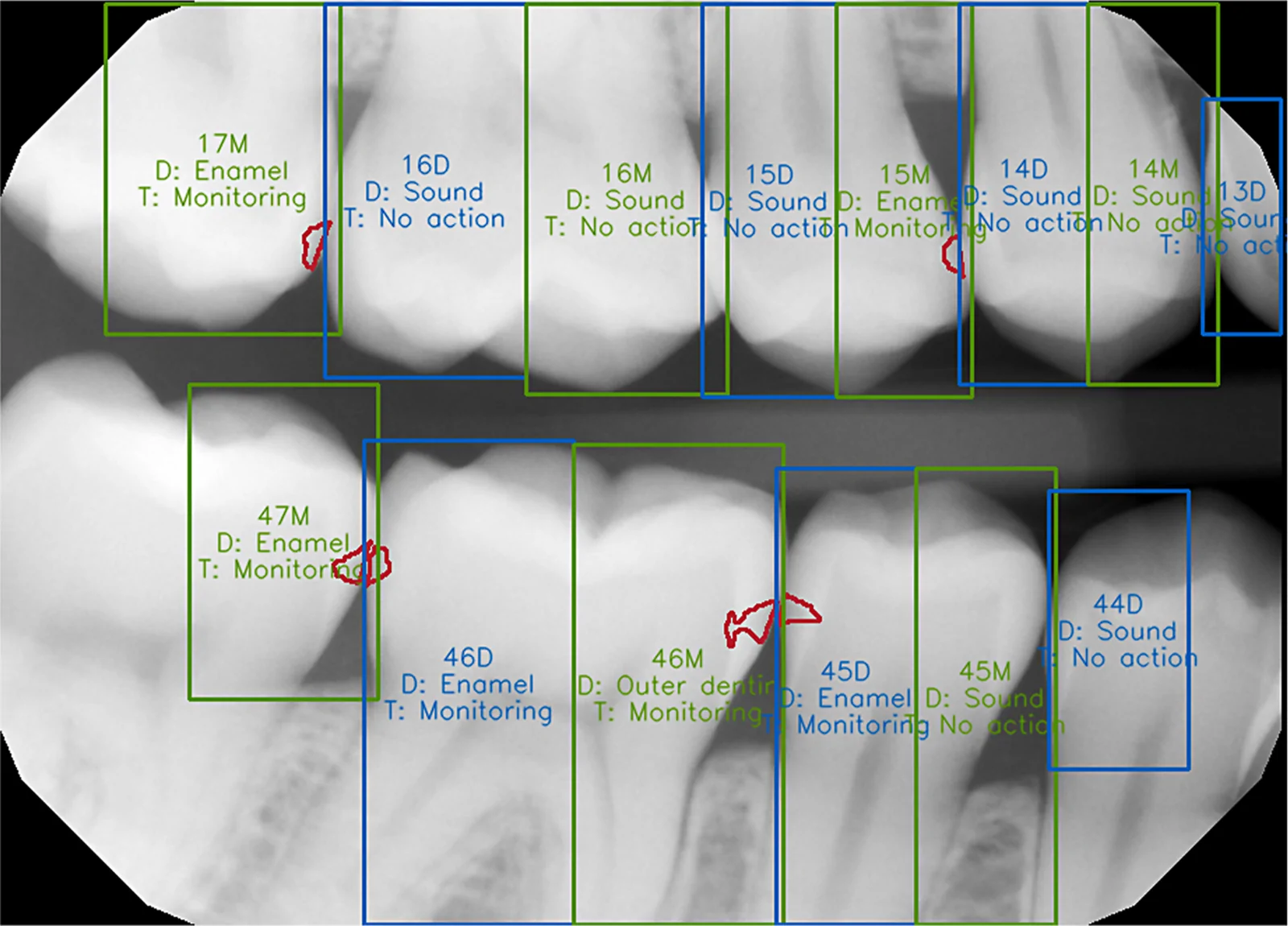
Example bitewing radiograph from the ACTA-Bw25-RefStd dataset showing the consensus reference-standard annotations. Red contours indicate the manually delineated carious regions. Blue and green rectangles denote the distal and mesial proximal surface regions, respectively. For each surface, the text displays the radiographic depth category (D) and the corresponding recommended treatment (T) as defined by the reference standard.

### Statistical Analysis

Table 2 reports the effect of AI support on radigraphic detection and staging accuracy for each professional group and for all observers combined. Odds ratios quantify whether detection and staging accuracy relative to the reference standard improved (OR > 1) or decreased (OR < 1) when AI support was used. Dental students showed a statistically significant improvement (OR = 1.209, *p* = 0.039). AI support had no statistically significant effect on detection and staging for general dentists (OR = 1.10, *p* = 0.28), dental radiologists (OR = 0.87, *p* = 0.15), and all observers combined (OR = 1.06, *p* = 0.31).

**Table 2.** Effect of AI support on radiographic detection and staging accuracy by observer group (CLMM)

Group	Odds ratio (95% CI)	*p* value
Dental students	1.21 (1.01–1.45)	0.04
General dentists	1.10 (0.92–1.36)	0.28
Dental radiologists	0.87 (0.73–1.05)	0.15
All	1.06 (0.95–1.17)	0.31

Post hoc simulation-based power analysis indicated adequate power for dental students (0.94) but limited power for general dentists (0.40) and dental radiologists (0.70); the full power calculation is detailed in online supplementary Material C.

### Observer Performance Metrics

#### Agreement Analysis (Quadratic-Weighted Cohen’s κ)

Table 3 reports quadratic-weighted Cohen’s κ values for lesion stage agreement with the reference standard in the without-AI and with-AI sessions and intra-observer agreement between both sessions. In the without-AI condition, dental radiologists show the highest agreement (0.75), while dental students show the lowest (0.59). With AI, general dentists and radiologists have comparable agreement (0.71 and 0.73), and AI itself reaches 0.67, reported for contextual comparison. In the intra-observer comparison, radiologists display the highest consistency (0.78), followed by dental students (0.77).

**Table 3.** Quadratic-weighted Cohen’s κ for caries lesion stage agreement across observer groups under the three proposed approaches

Observer	Without AI	With AI	Intra-observer
AI	–	0.67 (0.56–0.75)	–
Dental students	0.59 (0.49–0.70)	0.66 (0.62–0.71)	0.73 (0.68–0.78)
General dentists	0.71 (0.63–0.78)	0.71 (0.67–0.76)	0.70 (0.66–0.73)
Dental radiologists	0.75 (0.70–0.79)	0.73 (0.68–0.78)	0.78 (0.73–0.83)

Each cell shows the κ estimate, with corresponding 95% CI in parentheses.

#### Binary Detection Metrics

Table 4 presents the binary detection performance for all observer groups with and without AI support. Without AI support, radiologists showed the highest sensitivity (0.78), followed by general dentists (0.72) and dental students (0.59). With AI support, sensitivity decreased for radiologists (0.78 → 0.70) and general dentists (0.72 → 0.64), while students showed a small increase (0.59 → 0.60). Specificity was consistently high across groups in both sessions and improved for all groups when AI support was provided.

**Table 4.** Binary detection metrics for all observer groups under conditions with and without AI support

Session	Group	Sensitivity	Specificity	Balanced accuracy	PPV	NPV	F1 score
Without AI	Dental students	0.59 (0.46–0.71)	0.95 (0.91–0.98)	0.77 (0.71–0.83)	0.90 (0.84–0.95)	0.78 (0.72–0.83)	0.70 (0.60–0.79)
	General dentists	0.72 (0.59–0.83)	0.92 (0.87–0.97)	0.82 (0.77–0.86)	0.88 (0.82–0.94)	0.83 (0.77–0.88)	0.77 (0.70–0.83)
	Dental radiologists	0.78 (0.73–0.82)	0.91 (0.87–0.95)	0.84 (0.82–0.86)	0.87 (0.82–0.92)	0.86 (0.84–0.87)	0.82 (0.79–0.84)
With AI	Dental students	0.60 (0.54–0.68)	0.97 (0.94–0.99)	0.79 (0.76–0.82)	0.94 (0.89–0.98)	0.78 (0.75–0.81)	0.73 (0.68–0.79)
	General dentists	0.64 (0.58–0.68)	0.97 (0.96–0.99)	0.81 (0.78–0.83)	0.95 (0.92–0.97)	0.79 (0.77–0.81)	0.76 (0.72–0.79)
	Dental radiologists	0.70 (0.63–0.78)	0.94 (0.91–0.97)	0.82 (0.80–0.85)	0.90 (0.86–0.95)	0.82 (0.79–0.86)	0.78 (0.74–0.82)
​	AI tool	0.55 (0.47–0.63)	0.99 (0.96–1.00)	0.77 (0.73–0.81)	0.97 (0.92–1.00)	0.76 (0.71–0.81)	0.70 (0.63–0.77)

Values represent point estimates (95% CI).

PPV, positive predictive value; NPV, negative predictive value.

Balanced accuracy remained within a narrow range across all groups and sessions: students showed 0.77 without AI and 0.79 with AI; general dentists showed 0.82 without AI and 0.81 with AI; radiologists showed 0.84 without AI and 0.82 with AI. PPV remained high across groups, with values ranging from 0.87 to 0.95 across both sessions. NPV varied only modestly, ranging from 0.75 to 0.86 across groups. F1 scores followed the pattern observed for sensitivity: radiologists showed a decrease with AI support (0.82 → 0.78), general dentists showed a slight decrease (0.77 → 0.76), and students showed a slight increase (0.70 → 0.73).

For comparison, the standalone AI system demonstrated a sensitivity of 0.55 and very high specificity (0.99), resulting in a balanced accuracy of 0.77. This provides a reference point against which the influence of AI support on human observer performance can be interpreted.

Figure 2 displays two radar plots comparing binary detection metrics for dental students, general dentists, and dental radiologists for the session without AI support (left radar plot) and for the session with AI support (right radar plot). AI performance metrics were included in both plots to contextualize its influence on observer performance during the second session.

**Fig. 2.**
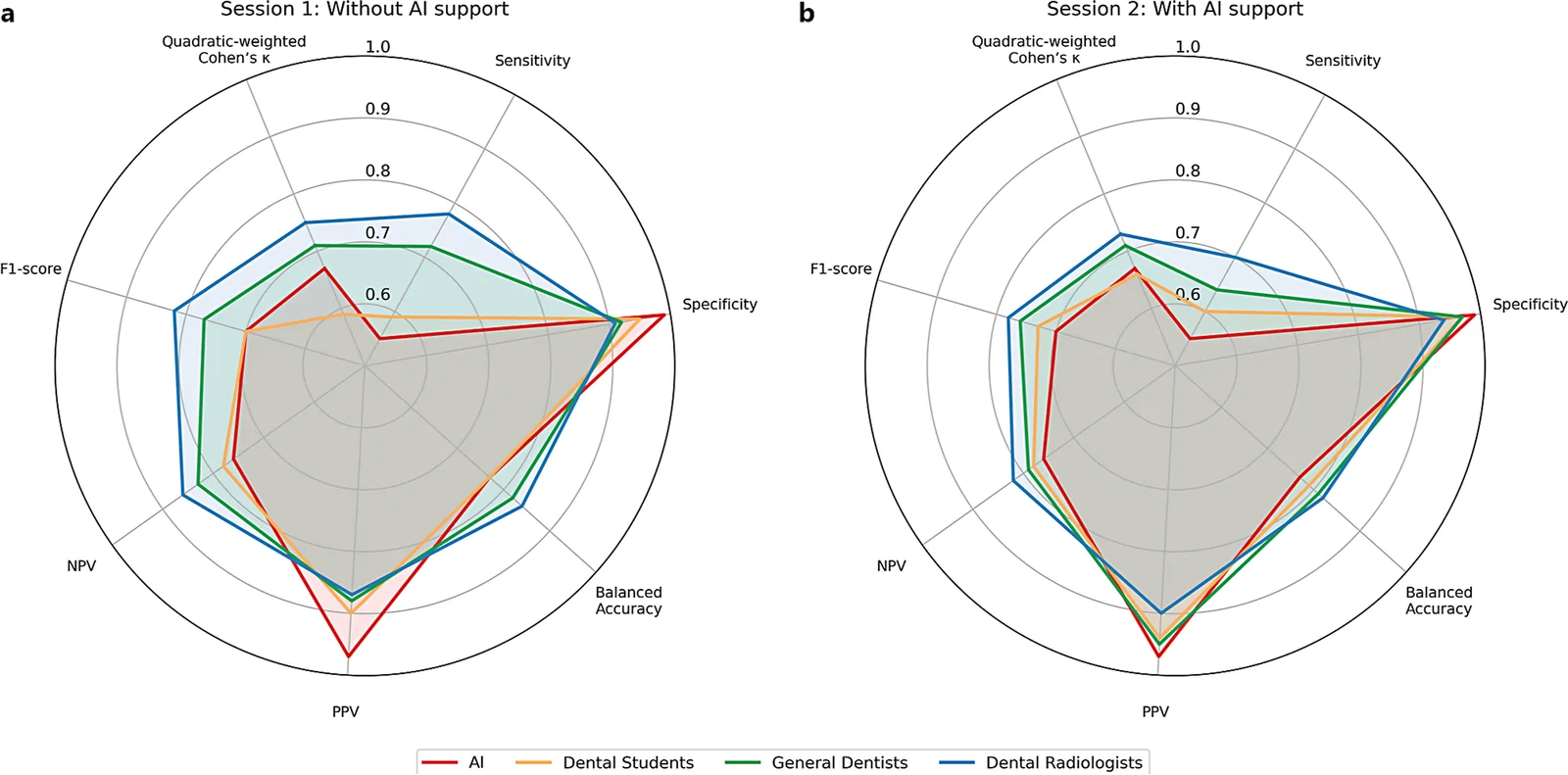
Radar plots summarizing performance metrics for each observer group and the standalone AI system. Plot (**a**) shows the performance metrics for the session without AI support. Plot (**b**) shows the performance metrics for the session with AI support.

Radar plots display several performance metrics simultaneously. The center reflects the lowest value shown, defined near the minimum observed performance, and the outer edge reflects the maximum attainable value for each metric (1.00).

Across both sessions, dental radiologists maintained the most stable and consistently high-performance profiles, showing strong values for most metrics, including sensitivity, balanced accuracy, NPV, F1 score, and quadratic-weighted Cohen’s κ (agreement). The standalone AI system exceeded all observer groups only in specificity and PPV, two metrics in which emphasis is on minimizing false positives, while underperforming in sensitivity-related measures.

When AI support was introduced, the performance shapes of dental students and general dentists shifted toward the characteristic profile of the AI system. This was most evident in the increases in specificity and PPV and the concurrent declines in sensitivity and F1 score. Quadratic-weighted Cohen’s κ values also showed modest adjustments consistent with these shifts. Dental radiologists exhibited comparatively minimal change, retaining a well-balanced profile with only subtle variations across metrics.

#### SR-Confidence Patterns across Lesion Stages

Figure 3 illustrates SR-confidence patterns across lesion stages for correct and incorrect assessments. Without AI support, all groups showed higher SR confidence for correct than for incorrect lesion stage assignments, at every assigned caries lesion stage diagnosed. With AI, SR confidence increased for both outcomes; among students and general dentists, this reduced the distinction between correct and incorrect assessments, whereas radiologists maintained clearer separation. In both sessions, incorrect assessments consistently showed wider CI.

**Fig. 3.**
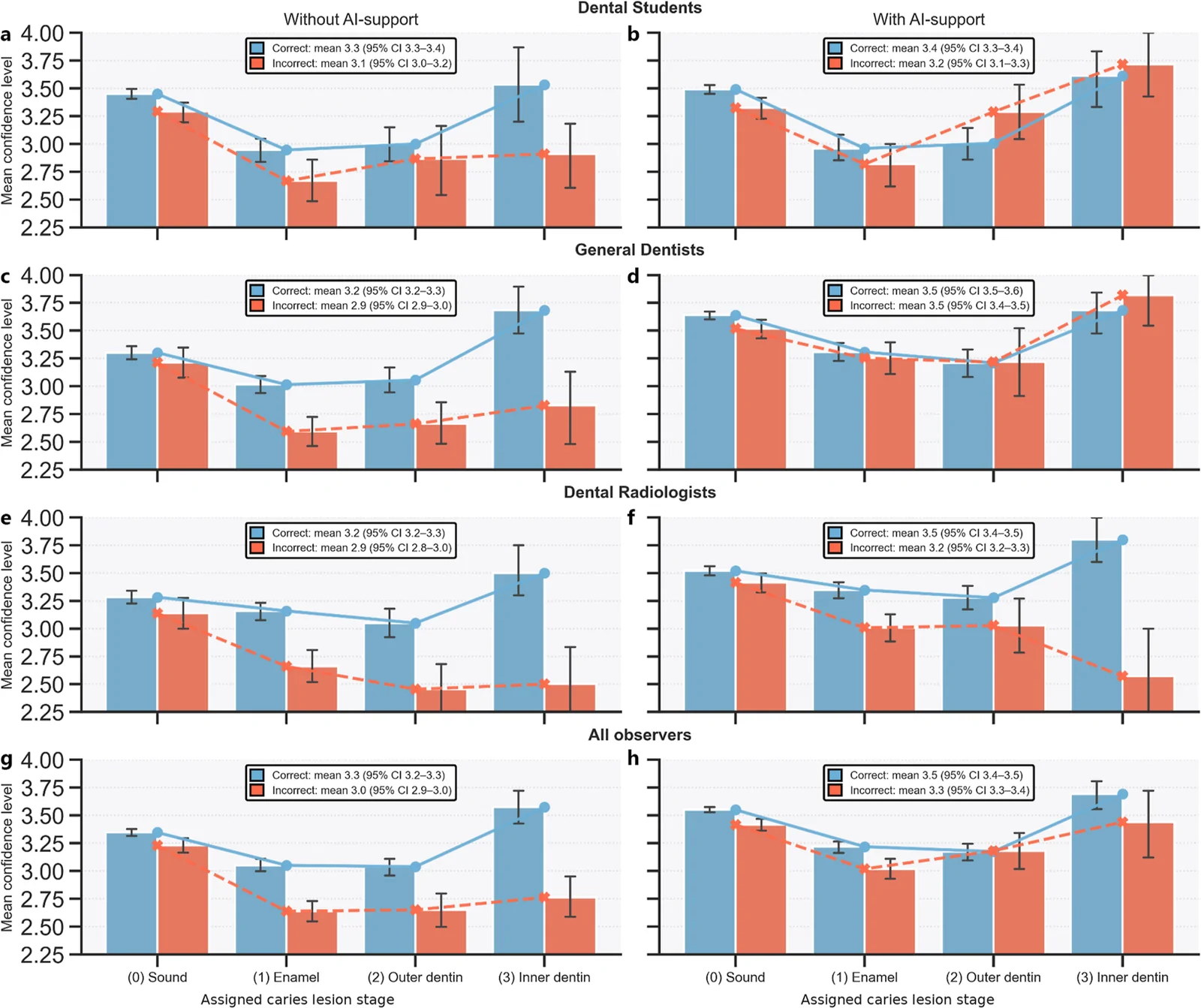
Mean confidence levels (95% CI) for correct (blue) and incorrect (red) stage assignments across assigned lesion stages, shown for sessions without (left column) and with (right column) AI support. The *x*-axis represents the assigned lesion stage, ordered from sound to inner dentin, while the *y*-axis refers to the SR confidence. Bars show mean confidence with 95% CIs, and transverse dashed lines show the pattern of confidence across assigned lesion stages. Panels (**a–h**) correspond to dental students without AI support (**a**) and with AI support (**b**), general dentists without AI support (**c**) and with AI support (**d**), dental radiologists without AI support (**e**) and with AI support (**f**), and all observers without AI support (**g**) and with AI support (**h**).

#### Session-Duration Analysis

Across the full sample, observers completed the assessment significantly faster when AI support was available (mean reduction: 12.3 min; Wilcoxon *p* = 0.0077). Dental students showed a statistically significant decrease in session duration (*p* = 0.0339). General dentists demonstrated a nonsignificant trend toward shorter sessions (*p* = 0.156). Dental radiologists exhibited a borderline reduction (*p* = 0.051). No observer required more time with AI support than without it, indicating a consistent direction of the effect across all groups. Detailed observer session durations and washout-period distributions are provided in online supplementary Material F.

## Discussion

This study shows that AI support influences proximal early caries evaluation in a measurable yet uneven manner. While it helped less experienced observers align more closely with the reference standard, it also increased SR confidence in both correct and incorrect stage assignments and shifted assessments toward the AI-based tool’s inherent tendencies across all observer groups. Overall, the results show that AI support improved efficiency and radiographic detection and staging accuracy for dental students, who completed their sessions significantly faster with AI support, while offering limited advantages, and possible constraints, for more experienced practitioners, among whom no statistically significant reductions in session duration were observed.

The literature reports heterogeneous outcomes for AI-assisted proximal caries detection, and our findings show areas of agreement as well as divergence from literature. A randomized clinical trial among general dentists reported clear sensitivity gains for enamel lesions under AI support [7]; whereas in our study, improvements emerged primarily for dental students and were expressed mainly as increased radiographic detection and staging accuracy, driven by higher specificity rather than sensitivity. This shift toward higher specificity implies a reduced likelihood of unnecessary restorative interventions, thereby providing a direct clinical benefit for patients. Another trial also demonstrated more efficient radiological interpretation behavior among dentists when AI annotations were present [8], an effect compatible with our results for students and, to a lesser extent, general dentists, whose performance also shifted toward higher specificity. Other investigations involving dental student training found no meaningful improvement in detecting early proximal enamel lesions, despite modest gains in positive agreement [17], similar to our observation that sensitivity did not improve for these students. Conversely, another dental student-oriented study reported more positive outcomes with an AI tool but noted that its model performed less reliably on enamel lesions, suggesting that perceived gains reflected the detection of deeper lesions rather than improvement in early-stage caries detection [18]. Because our study specifically targeted early proximal lesions, a domain where both clinicians and current AI models have reduced sensitivity, we can conclude that consistent with the literature, AI support yields limited benefit for early-lesion detection [1, 14, 19, 20].

The current study presents several strengths. First, its fully crossed design enabled evaluation of every observer under both conditions, with and without AI support, allowing the specific influence of AI assistance to be isolated. Second, the use of a robust reference standard derived through expert consensus strengthens the interpretation of radiographic detection and staging accuracy outcomes. Third, the public availability of this reference standard enhances transparency and facilitates future comparative studies. Fourth, the session protocol was carefully designed to minimize cross-session bias and to avoid any influence of one condition on the other. Fifth, inclusion of SR-confidence ratings provides insight into the degree of comfort and potential risks associated with using AI support. Sixth, the participation of dental students, general dentists, and dental radiologists offers a broader view of how AI influences clinicians with different levels of experience.

Several limitations should also be acknowledged. First, although a rigorous reference standard was constructed, it does not constitute a true gold standard because expert radiographic assessments cannot be assumed to be error free given the inherent limitations of bitewing imaging, and this absence of an error-free gold standard inherently affects estimates of radiographic detection and staging accuracy. Therefore, the reported accuracy should be interpreted as the agreement with the reference standard rather than as absolute clinical accuracy. Second, patient-level information, such as caries risk, symptoms, or clinical findings, was not available. Third, observers represented varying experience levels and gender, and observers were affiliated with ACTA, introducing potential institutional bias. In addition, although observer groups were defined by professional role, clinical experience varied within the general dentist and dental radiologist groups. This included two general dentists with only 1–2 years of clinical experience and two dental radiologists with more years of clinical experience than some members of the expert panel, which should be considered when interpreting group-level comparisons. Fourth, some participants reported recommending restorations for clinically “sound” surfaces due to perceived defects in existing fillings, which may complicate interpretation of detection outcomes due to differences in participant opinions. Fifth, although the study included only 25 bitewing radiographs, the proximal surface was the unit of analysis. Therefore, the analyses were based on 369 measures, which were evaluated by all observers in both sessions. Nevertheless, the limited number of observers per group should be recognized, particularly because simulation-based power analyses indicated limited power for the general dentists and dental radiologists. Sixth, this study showed that dental students were influenced by the suggestions of the AI-based tool and suggests that a similar effect may apply to other participant groups. However, compared with manual observations, the AI-based tool detected fewer subtle early proximal caries lesions, which limits its supportive value in these cases. Seventh, although the patterns in SR confidence appeared coherent, such subjective measures are inherently variable; incorporating more rigorous or objective approaches (e.g., repeated measurements or reliability testing) to assessing detection certainty in future work would help mitigate this limitation. Eighth, although radiographs with insufficient diagnostic quality were excluded, some overlap of proximal surfaces remained in selected images, reflecting common limitations of clinical bitewing radiography. This overlap may have reduced the reliability of early proximal caries detection, particularly for subtle enamel lesions. Ninth, power analyses indicated adequate power only for the student group, whereas power was limited for more experienced observers (see online suppl. Material C). Therefore, nonsignificant findings were observed in these latter groups. Finally, only one AI tool was evaluated, limiting generalizability to other tools with different detection performance profiles.

Several mechanisms likely underpin the positive influence of AI support observed in this study. First, the consistent shift in detection and staging tendencies, especially among dental students and general dentists, indicates cognitive anchoring to AI suggestions, whereby algorithmic cues shape the observer’s initial judgment and subsequently constrain further perceptual evaluation. As the AI-based tool used here achieved higher specificity but lower sensitivity than the observers, this anchoring mechanism plausibly explains why the only consistent performance gain across groups occurred in specificity. Second, AI annotations may have encouraged observers to trust surfaces flagged as healthy, streamlining decision-making and likely contributing to the shorter session times demonstrated for students, and possibly for the other groups (no significant improvements), while also potentially reducing scrutiny of subtle early enamel lesions that were not highlighted. This mechanism aligns with prior evidence that AI guidance can narrow visual search to annotated regions [8]. Third, our study suggests that the AI-support impact varied by experience level, with radiologists being less influenced despite increased SR confidence. A reasonable speculation is that, if AI-based tools can be trained to achieve performance at least comparable to experienced radiologists, or even surpass them in case a true gold standard becomes available, dental professionals may continue to align their decisions with the AI profile. In such a scenario, this alignment could enhance radiographic detection and staging accuracy and help reduce diagnostic variability. Whether these patterns would change when evaluated against a true gold standard remains an important open question.

The current results have several implications for the integration of AI into radiographic caries detection. First, AI-based tools may help less-experienced clinicians approach expert-level detection and staging patterns, but this benefit comes with the risk of aligning with the AI-based tool’s detection profile inaccuracies. Second, the observation that SR confidence increased even in incorrect assessments underscores the need for AI interfaces that convey uncertainty rather than offering only categorical outputs. This pattern aligns with participants’ comments, which frequently referenced assessment uncertainty arising from image quality limitations, overlap, and the absence of clinical context, as documented in online supplementary Material G. Third, anchoring effects demonstrate that clinicians should remain vigilant when interpreting AI-assisted radiographs, particularly for subtle early lesions that AI may under-detect. Fourth, AI-based tools should also incorporate patient-level caries risk factors, consistent with evidence-based caries management and safe radiographic practice, since clinicians diagnose patients, not images [21, 22].

For clinical implementation, several recommendations can be made. First, a robust and universally accepted reference standard should be established. Developers should be required to evaluate their AI-based tools against this benchmark to produce a transparent performance profile that helps clinicians understand each tool’s strengths and limitations and decide whether and how it should be used. Second, clinician decision-making may be further enhanced if AI-based tools provide not only lesion detections but also pixel-level confidence maps, allowing users to exercise greater caution when the tool signals low certainty. Third, these AI-based tools would become more clinically valuable if they delivered not just lesion localization but also staging information, thereby offering clearer guidance for subsequent treatment decisions. Altogether, these recommendations highlight the importance of integrating AI-based tools in ways that preserve clinicians’ clinical autonomy through interfaces that communicate uncertainty, training that emphasizes critical appraisal of AI outputs, and continued refinement to ensure reliable performance for subtle proximal early caries lesions.

## Conclusion

This study demonstrates that AI support alters clinicians’ radiographic detection and staging decisions by aligning judgments with the AI detection profile. The effect of AI support on radiographic detection and staging accuracy varied across observer groups, with no statistically significant changes observed in the more experienced groups. The increases in diagnostic SR confidence, even for incorrect decisions, underscore the need for evaluation frameworks that extend beyond algorithmic performance to include observer-AI interaction effects in proximal early caries assessment.

## Acknowledgments

We sincerely thank the 21 participating dental professionals, including dentistry students, general dentists, and dentists with radiology background, for their valuable time when participating in this study. We are grateful to Michael Tanck (Amsterdam UMC, Epidemiology and Data Science [EDS]) for his expert guidance and assistance with the statistical methodology. Editorial assistance was provided by ChatGPT for phrasing and clarity only; no factual content was generated by the tool.

## Statement of Ethics

All procedures involving anonymized radiographic data were conducted in accordance with the ethical standards of the Academic Centre for Dentistry Amsterdam (ACTA). The ACTA Institutional Review Board reviewed and approved the study protocol under approval No. 2025-37060. No personally identifiable information was used, and all data were handled in accordance with institutional and GDPR guidelines. Written informed consent was obtained from all participants prior to participation in the study.

## Conflict of Interest Statement

The authors have no conflicts of interest to declare.

## Funding Sources

This study was not supported by any sponsor or funder.

## Author Contributions

R.E.G.-V., Q.T.D.V., H.M., and E.B. conceived the study. R.E.G.-V., Q.T.D.V., P.M., B.G.L., H.M., and E.B. designed the methodology, interpreted the findings, and drafted the manuscript and approved the final version. R.E.G.-V., Q.T.D.V., and E.B. acquired the data. R.E.G.-V., Q.T.D.V., P.M., H.M., and E.B. performed the analysis.

## Data Availability

The ACTA-Bw25-RefStd dataset used in this study is publicly available in the Vrije Universiteit Amsterdam Dataverse repository at https://doi.org/10.48338/VU01-UHGI3K. Researchers who use this dataset are kindly asked to cite this publication as the main reference. Further inquiries can be directed to the corresponding author.
